# Drug coated balloon improves outcomes of sub-optimal Supera deployment in the intermediate term

**DOI:** 10.1038/s41598-022-25634-z

**Published:** 2022-12-09

**Authors:** Prakash Krishnan, Raman Sharma, Sriya Avadhani, Arthur Tarricone, Allen Gee, Serdar Farhan, Haroon Kamran, Annapoorna Kini, Samin Sharma

**Affiliations:** Department of Medicine/Cardiology, Icahn School of Medicine at Mount Sinai, The Zena and Michael A. Wiener Cardiovascular Institute and the Marie-Josée Henry R. Kravis Cardiovascular Health Center, Icahn School of Medicine at the Mount Sinai Hospital, 1 Gustave L. Levy Place, Box 1030, New York, NY 10029 USA

**Keywords:** Cardiology, Interventional cardiology

## Abstract

Sub-Optimal deployment of Self expanding interwoven stents (Supera) has been shown to increase the rate of Clinically Driven Target Lesion Revascularization (CD-TLR). Meanwhile, drug coated balloons (DCB) have been shown to reduce CD-TLR in the femoral-popliteal segment in de- novo and restenotic lesions. However, the clinical effects of vessel preparation with DCB in nominal, compressed, and elongated Supera has not been widely studied. The purpose of this study is to assess the outcomes of clinically driven re-intervention, major amputations, and mortality in relation to the use of DCB as vessel preparation in different deployment conditions (nominal, compressed, elongated) of the Supera stent. Patient chart data was collected at a single center hospital between March 2015 and May 2020. All patients were adults (≥ 18 years old) and were treated with at least one (≥ 1) Supera stent. Deployment status was extrapolated from anonymized angiograms. The primary endpoint of this study was CD-TLR. Secondary endpoints included amputation and mortality rates associated with each deployment condition. A total of 670 limbs were treated and patients were followed for 36 months. Nominal stent deployment was observed in 337 limbs, followed by elongated condition (n = 176), then by compressed conditions (n = 159). CD-TLR was observed most frequently with elongated deployment. Drug coated balloons were used as vessel prep prior to stenting and showed a protective effect regardless of deployment status; O.R = 0.44 (CI 0.30–0.66, *p* < .05).

## Introduction

Endovascular treatment of lower extremity peripheral artery disease (PAD) has been proven safe and efficacious and has become the preferred treatment over surgical management due to multiple benefits^1^. Current guidelines recommend endovascular therapy for superficial femoral artery disease as the first line approach for trans -Atlantic-inter-society consensus (TASC) A-C lesions and select TASC D lesions^2^. Despite advances made in endovascular management of PAD, durability of clinical results remains suboptimal^3–5^. Supera stents are known for their flexibility, ability to withstand greater femoropopliteal arterial stress, and have also exhibited reduced vessel restenosis and target lesion revascularization when compared to percutaneous transluminal angioplasty^6^. Long term outcomes utilizing the Supera stent design have demonstrated promising results out to 36 months; however variation exists depending on deployment conditions. In the Superb trial, elongated deployment was associated with increased CD-TLR and decreased patency, likely attributed to neointimal hyperplasia from shear stress^7^.

Paclitaxel drug coated balloons (DCB) have been shown to reduce neointimal hyperplasia and improve both patency and CD-TLR compared to plain old balloon angioplasty (POBA)^8–10^. The aim of this study is to investigate the effects of DCB use as vessel prep prior to Supera stenting in the intermediate term across various Supera deployment status (compressed, nominal and elongated).

## Methods

Between March 2015 to March 2020, patients treated with Supera interwoven nitinol stents at a single center were identified. All patients were included in the study if they were treated with at least one (≥ 1) Supera stent (Supera peripheral stent Abbott Laboratories, IL, USA). The treatment modality and selection of devices as well as the duration of dual antiplatelet therapy after endovascular intervention were under discretion of the treating physician. All patients were treated with post procedure medical therapy in accordance with standard institutional practice of Plavix-clopidogrel (75 mg/day), and aspirin (81 mg) for 3 months. All lesions were pre-dilated with either DCB or PTA at the discretion of the operator. All patients were followed in a Standard institutional surveillance program, consisting of routine duplex ultrasonography at 1,3,6 and 12 months as well as clinical follow up at the same time frame. The study has been approved by the local governing Institutional Review Board of the Mount Sinai School of Medicine: Program for Protection of Human Subjects committee. All study methods were carried out in accordance with relevant guidelines. An informed consent waiver was provided by the ethics committee at the Institutional Review Board of the Mount Sinai School of Medicine: Program for Protection of Human Subjects committee as this study is retrospective/ observational in design and utilized FDA approved devices.

The primary outcome of this study was freedom from clinically driven-target lesion revascularization (CD-TLR), defined as freedom from any same target lesion re-intervention event post index procedure, and was determined by duplex ultrasonography or clinical symptomatology assessed by the physician. Secondary endpoints consisted of amputation free survival (inclusive of both major, above-the-knee and minor, below-the-knee amputations) and mortality by latest follow-up visit. Angiograms were reviewed for lesion morphology, reference vessel diameter, dissection, and deployment condition (compression, nominal, elongation) by 2 physicians independently. Disagreements were resolved by the corresponding author. All angiograms were anonymized prior to review by the data collection team. Reference vessel diameter was established through quantitative angiography. The estimation of compression and elongation was determined by the operator, by dividing the nominal stent length according to package insertion directions. Actual deployment length was measured using a preprocedural attached measuring scale. Optical evaluation of the complete stent was then conducted to examine for signs of elongation or compression. In keeping with the findings in the SUPERB trial^7^ the authors define the final three Supera stent dimensions as elongation (≥ 10% of package insert length), compression (≤ 10% of package insert length), and nominal (− 10% ≤ Length ≤  + 10% of package insert length). 10% was chosen due to the increase in CD-TLR observed in the SUPERB trial^7^ when the Supera deployment was either compressed or elongated greater than 10%.

Continuous variables were presented as mean ± standard deviation and categorical variables were presented as n (n/total). Shapiro–Wilk test was used to determine normality (n ≤ 50) while Kolmogorov–Smirnov test was used for entire cohort variables (n ≥ 50). All statistical analyses were completed using SAS version 9.3 (SAS, Cary, North Carolina). Univariate and multivariable Cox Proportional Hazards Regression models were used to determine the predictors of CD-TLR, amputation, and mortality. For the multivariable analyses, CD-TLR , amputation and mortality were censored at 1095 days. Candidate variables for the multivariable models were selected based on: (1) *P* < 0.20 on the univariate analysis; (and clinically relevant, procedurally actionable variables. Statistical significance was indicated by *p*-values ≤ 0.05. All laboratory and demographic information were determined from EPIC patient medical records and procedural characteristics were extracted from catherization reports. The datasets generated and/or analyzed during the current study are not publicly available due to patient confidentiality but are available from the corresponding author on reasonable request.

## Results

A total of 672 limbs were treated between 2015 and 2020 at the level of the popliteal or superficial femoral arteries. There were 312 males and 360 females included in this study, with an average subject age of 71.2 years (Table 1). Further baseline descriptive characteristics between Supera and Supera + DCB treated subjects are presented in Table 2. Deployment conditions were of the following: nominal, elongated, and compressed. 585 limbs were treated with a single Supera stent, while 87 limbs were treated with > 1 Supera stents. In the case of the 87 limbs treated with > 1 Supera stents, total stent length was used in order to determined compressed, nominal, or elongated. After angiographic review of deployment condition 3/672 cases were not universally agreed upon. The corresponding author analyzed the cases with the reviewers and consensus was agreed upon. Nominal was the most frequently deployed condition (n = 337, 133 Supera, 204 Supera + DCB), followed by elongated (n = 176, 50 Supera, 109 Supera + DCB), compressed (n = 159, 53 Supera, 123 Supera + DCB) (Table 2).

**Table 1:** Baseline characteristics.

	Nominal	Compressed	Elongated	*p*-value
n	337	157	176	
Age	71.21 ± 10.11	71.32 ± 10.43	71.54 ± 10.33	0.90
Reference Vessel Diameter	5.46 ± 0.694	5.57 ± 0.464	5.46 ± 0.62	0.07
Lesion Length (mm)	92.22 ± 39.40	96.80 ± 39.61	84.71 ± 37.60	0.06
Chronic Kidney Disease	10 (3.0%)	5 (3.2%)	5 (2.8%)	0.98
Coronary Artery Disease	214 (63.5%)	106 (67.5%)	137 (77.8%)	0.68
Diabetes Mellitus	231(68.5%)	106 (67.5%)	98 (55.7%)	0.53
Smoker	254 (75.4%)	123 (78.3%)	135 (76.7%)	0.76
Hypertension	328 (97.3%)	150 (95.5%)	173 (98.3%)	0.35
Hyperlipidemia	317 (94.1%)	147 (93.6%)	169 (96.0%)	0.51
Critical Limb Ischemia	59 (17.5%)	25 (15.9%)	32 (18.2%)	0.85
Calcification	63 (18.7%)	30 (19.1%)	38 (21.6%)	0.74
**Rutherford Score**				
1	0 (0.0%)	0 (0.0%)	0 (0.0%)	N.A
2	15 (4.4%)	13 (8.3%)	3 (1.7%)	0.74
3	212 (62.9%)	105 (66.9%)	116 (65.9%)	0.93
4	55 (16.3%)	16 (10.2%)	26 (14.8%)	0.81
5	47 (13.9%)	21 (13.3%)	28 (15.9%)	0.91
6	8 (2.4%)	2 (1.3%)	3 (1.7%)	0.89

**Table 2:** Supera versus Supera + DCB Baseline Characteristics.

	Supera + PTA	Supera + DCB	*p*-value
n	236	436	
Age	71.73 ± 10.22	71.10 ± 10.21	0.42
Reference Vessel Diameter	5.45 ± 0.84	5.54 ± 0.48	0.43
Chronic Kidney Disease	5 (2.1%)	15 (3.4%)	0.31
Coronary Artery Disease	181 (76.7%)	253 (58.0%)	< 0.05
Diabetes Mellitus	161 (68.8%)	313 (71.8%)	0.42
Smoker	177 (75.6%)	335 (76.8%)	0.73
Hypertension	228 (97.4%)	423 (97.0%)	0.75
Hyperlipidemia	221 (94.4%)	412 (94.5%)	0.99
Critical Limb Ischemia	46 (19.7%)	70 (16.1%)	0.25
Directional Atherectomy	164 (70.1%)	305 (70.0%)	0.91
Laser Atherectomy	33 (14.1%)	87 (20.0%)	0.88
Jet Stream	37 (15.8%)	44 (10.1%)	0.93
Nominal	133 (56.8%)	204 (46.8%)	0.01
Compressed	50 (21.4%)	107 (24.5%)	0.29
Elongated	53 (22.6%)	123 (28.2%)	0.11
Supera Stent Length (mm)	92.18 ± 36.75	90.83 ± 40.44	0.67
**Rutherford score**			
1	0 (0.0%)	0 (0.0%)	N.A
2	10 (4.3%)	21 (4.8%)	0.91
3	138 (59.0%)	295 (67.7%)	< 0.05
4	40 (17.1%)	57 (13.1%)	0.20
5	40 (17.1%)	56 (12.8%)	0.17
6	6 (2.6%)	7 (1.6%)	0.57

Primary and each secondary outcome, comorbidities, and general lesion characteristics in reference to each deployment condition are presented in Tables 3, 4 and 5. Drug coated balloons (DCB) were used as vessel prep in 436/672 (65%) procedures. All patients in the Supera + DCB group received the IN.PACT Admiral DCB ( Medtronic). Of the subjects treated with DCB, deployment conditions varied with compression (n = 109), nominal (n = 204), and elongated (n = 123). The frequency of CD-TLR was significantly lower in DCB treated subjects compared to non-DCB treated subjects across all deployment conditions (Tables 3, 4 and 5). Multivariable analysis suggests that DCB use as vessel preparation prior toSupera deployment is protective of CD-TLR regardless of deployment status (OR 0.35; CI 0.26–0.47, *p* < 0.05).Amputation and mortality rates were similar within each deployment cohort despite the presence or absence of DCB utility.

**Table 3:** Nominal deployment outcomes with and without DCB.

	DCB n = 204	NO DCB n = 133	*p*-value
CD-TLR	16 (7.8%)	23 (17.3%)	< 0.01
Amputation	10 (4.9%)	6 (4.5%)	0.87
Mortality	8 (3.9%)	6 (4.5%)	0.79
Gender (Male)	105 (51.4%)	76 (57.1%)	0.44
CKD	6 (2.9%)	4 (3.0%)	0.97
DM	141 (69.1%)	90 (67.7%)	0.78
HTN	199 (97.5%)	129 (97.0%)	0.76
HLD	190 (93.1%)	127 (95.5%)	0.37
CLI	35 (17.2%)	24 (18.0%)	0.83
Dissection	115 (56.4%)	43 (32.3%)	< 0.01
Calcification	28 (13.7%)	35 (77.4%)	< 0.01
CTO	73 (35.8%)	53 (39.8%)	0.58
Perforation	4 (2.0%)	1 (1.0%)	0.37
**Rutherford**			
1	0 (0.0%)	0 (0.0%)	N.A
2	10 (4.3%)	5 (3.8%)	0.57
3	132 (64.7%)	80 (60.2%)	0.46
4	31 (15.2%)	24 (18.0%)	0.59
5	27 (13.2%)	20 (15.0%)	0.76
6	4 (2.0%)	4 (3.0%)	0.80

**Table 4:** Compressed deployment outcomes with and without DCB.

	DCB n = 107	NO DCB n = 50	*p*-value
CD-TLR	10 (9.3%)	12 (24.0%)	0.01
Amputation	7 (6.5%)	4 (8.0%)	0.76
Mortality	4 (3.7%)	2 (4.0%)	0.92
Gender (Male)	61 (57.0%)	36 (72.0%)	0.05
CKD	5 (4.7%)	0 (0.0%)	0.12
DM	74 (69.2%)	34 (68.0%)	0.99
HTN	104 (97.2%)	48 (96.0%)	0.87
HLD	103 (96.3%)	45 (90.0%)	0.30
CLI	18 (16.8%)	9 (18.0%)	0.82
Dissection	48 (44.9%)	19 (38.0%)	0.47
Calcification	21 (19.6%))	10 (20.0%)	0.91
CTO	44 (41.1%)	22 (44.0%)	0.67
Perforation	4 (3.7%)	1 (2.0%)	0.56
**Rutherford**			
1	0 (0.0%)	0 (0.0%)	N.A
2	8 (7.5%)	5 (10.0%)	0.50
3	75 (70.1%)	30 (60.0%)	0.28
4	10 (9.3%)	6 (12.0%)	0.82
5	14 (13.1%)	7 (14.0%)	0.92
6	2 (1.9%)	0 (0.0%)	0.78

**Table 5:** Elongated deployment outcomes with and without DCB.

	DCB n = 123	NO DCB n = 53	*p*-value
CD-TLR	33 (26.8%)	28 (52.8%)	< 0.01
Amputation	13 (10.6%)	9 (17.0%)	0.24
Mortality	6 (4.8%)	3 (5.7%)	0.83
Gender (Male)	54 (43.9%)	29 (54.7%)	0.19
CKD	4 (3.3%)	1 (1.9%)	0.62
DM	98 (79.7%)	39 (73.6%)	0.37
HTN	120 (97.6%)	53 (100.0%)	0.25
HLD	119 (96.7%)	50 (94.3%)	0.45
CLI	17 (13.8%)	15 (28.3%)	0.02
Dissection	61 (49.6%)	27 (50.9%)	0.87
Calcification	22 (17.9%)	16 (30.2%)	0.07
CTO	39 (31.7%)	21 (39.6%)	0.31
Perforation	1 (0.8%)	0 (0.0%)	0.51
**Rutherford**			
1	0 (0.0%)	0 (0.0%)	N.A
2	3 (2.4%)	0 (0.0%)	0.81
3	88 (71.5%)	28 (52.8%)	< 0.05
4	16 (13.0%)	10 (18.9%)	0.44
5	15 (12.2%)	13 (24.5%)	0.07
6	1 (0.8%)	2 (3.8%)	0.45

Multivariable sub-analysis incorporating each deployment condition (compressed, nominal, elongated) in the presence or absence of DCB revealed protective effects in DCB as well: nominal (O.R = 0.41 (CI 0.27–0.62, *p* < 0.05), compressed (O.R = 0.32 (CI 0.13–0.80, *p* < 0.05) and elongated (O.R = 0.33 (CI 0.17–0.64, *p* < 0.05) (Table 6). In the elongation deployed group specifically, an average of 3.8 subjects would need to receive DCB in order to prevent one reintervention event.

**Table 6:** DCB associations with reintervention grouped by deployment condition.

Deployment condition	Odds ratio	Confidence Interval	*p*-value
Nominal	0.41	0.27–0.62	< 0.05
Compressed	0.32	0.13–0.80	< 0.05
Elongated	0.33	0.17–0.64	< 0.05

**Figure 1:**
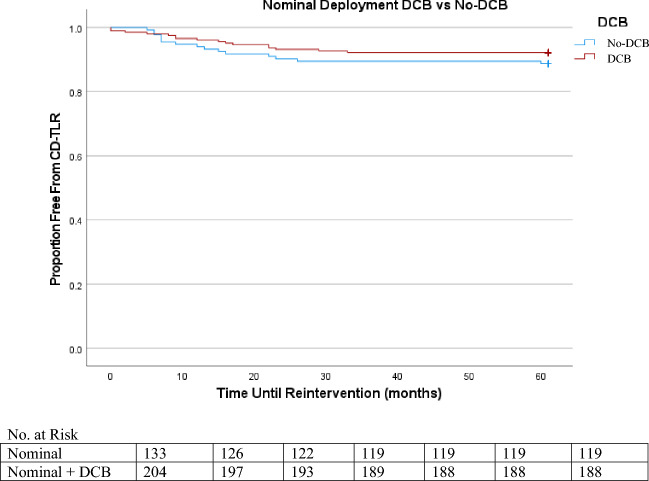
Nominal DCB versus Nominal No-DCB K-M CD-TLR.

**Figure 2:**
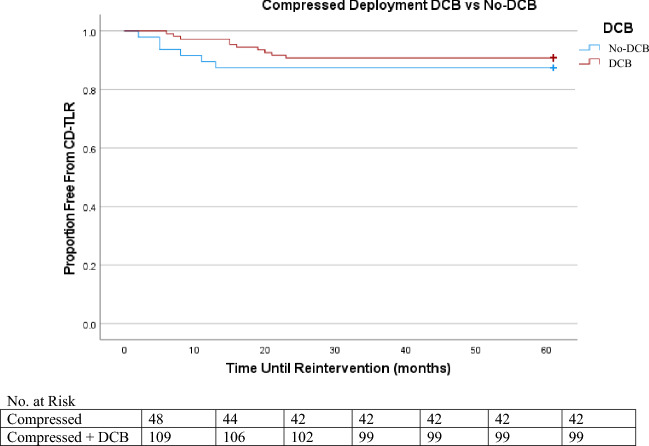
Compressed DCB versus Compressed No-DCB K-M CD-TLR.

**Figure 3:**
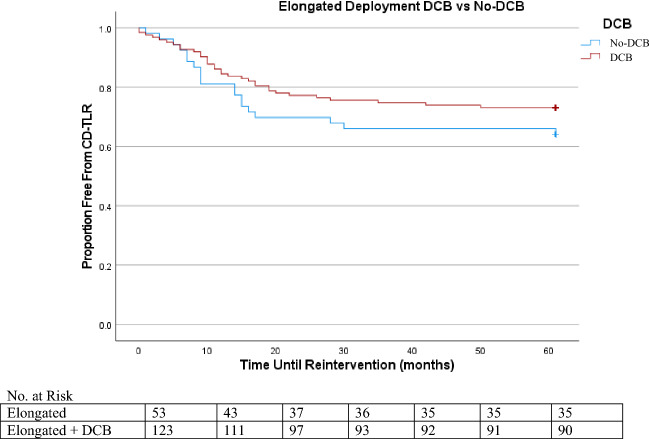
Elongated DCB versus Elongated No-DCB K-M CD-TLR.

In Kaplan Meier, the lowest freedom from reintervention was elongated deployment, followed by nominal, then by compressed (Figures 1, 2 and 3). By the termination of the study, 91% of the nominal sub-group, 90% of the compressed sub-group, and 70% of the elongated sub-group were free from CD-TLR. Of the entire cohort, freedom from CD-TLR at 36 months was 82.5% (553/670).

## Discussion

In the present study we assess the outcomes of CD-TLR, amputation, and mortality associated with different deployment conditions of the Supera stent, and the outcomes associated with DCB used as vessel preparation.

Multivariable analysis suggests that DCB used prior to Supera deployment is protective of CD-TLR regardless of deployment status (OR 0.35; CI 0.26–0.47, *p* < 0.05).

A proposed benefit of the Supera is the helical design does not apply chronic outward force theorized to contribute to In stent restenosis. Despite this improvement in design, CD-TLR in Supera has varied across multiple studies with rates ranging between 55 and 90%^7,11,12^. A plausible explanation for the variance in outcomes may be deployment condition of the Supera. Multiple factors affect accurate Supera deployment including lesion characteristics , adequate vessel prep , proper Supera sizing, level of operator experience and the innate technical challenges associated with Supera deployment^6^. Angiographically adequate vessel preparation and sizing were visualized, and > 50% of the Supera were deployed correctly. Sub-optimal deployment has been shown to occur in greater than 1/3^rd^ of procedures in previous studies^7,13^. Subsequently, this mal-deployment increases the risk for intimal hyperplasia, dissections, ruptures and ultimately reduced patency and increased CD-TLR^7,13,14^.

DCB in addition to Supera has been studied in a randomized , prospective, multicenter study with no significant differences found in 1 year primary patency, CD-TLR, amputation events, mortality or functional outcomes^15^. While these results suggested no added value to the addition of DCB to the primary stenting strategy; outcomes based on deployment conditions were not reported. Additionally , the prior study used a DCB that has not been studied in the US population and is not currently approved by the Federal Drug Administration(FDA). Similarities between the DCB(LEGFLOW) used in the RAPID study and the DCB (INPACT) used in this study include the paclitaxel dosage (3.5micorgrams/mm^2^) and a significant difference is the excipient (Urea ‘INPACT’ and Ammonium Salt solution LEGFLOW). This may explain the variation in results between the two studies and further illustrate that all DCB platforms should be evaluated individually. Previously, the SUPERB study reported decreased primary patency and CD-TLR in elongated Supera (74%) compared to nominal (91%) and compressed (74%)^7^. In the current study, DCB use as vessel preparation prior to deployment of Supera may reduce the CD-TLR in the event of a sub-optimal deployment regardless of deployment condition and may be prudent in the event of a sub optimal deployment of Supera^7^.

A limitation inherent to this study is having a retrospective design. Considering the data was collected through electronic health records and angiograms dating over 5 years, the investigators were unable to control for confounding factors that may have impacted the outcomes. This study was also limited to a single center teaching hospital, indicating that there has not been a replication of similar findings in different population groups. Additionally INPACT DCB was the only DCB used and limits generalizability of the current findings to other approved DCBs.

## Conclusion

Supera stents have shown promising results in endovascular therapy, especially for longer, complex lesions. However, stent deployment remains a difficult variable to control due to the number of factors that influence success. Vessel preparation with DCB was found to provide a protective effect in reintervention, regardless of deployment status.

## Perspectives

The Supera stent is designed to manage complex, femoropopliteal lesions; however, general patency of the stent is influenced by deployment success. Since deployment is dependent on numerous factors, mal-deployment is a common adverse event that can impact various clinical outcomes including revascularization rates of the target lesion. This study suggests that vessel preparation with DCB may reduce CD-TLR regardless of deployment status.

## Data Availability

The datasets generated and/or analyzed during the current study are not publicly available due to patient confidentiality but are available from the corresponding author on reasonable request.

## Abbreviations

PAD: Peripheral artery disease
CD-TLR: Clinically driven target lesion revascularization
DCB: Drug coated balloon
